# Effects of local anesthesia of the cerebellum on classical fear conditioning in goldfish

**DOI:** 10.1186/1744-9081-6-20

**Published:** 2010-03-23

**Authors:** Masayuki Yoshida, Ruriko Hirano

**Affiliations:** 1 Graduate School of Biosphere Science, Hiroshima University, Higashihiroshima 739-8528, Japan

## Abstract

**Background:**

Besides the amygdala, of which emotion roles have been intensively studied, the cerebellum has also been demonstrated to play a critical role in simple classical fear conditioning in both mammals and fishes. In the present study, we examined the effect of local administration of the anesthetic agent lidocaine into the cerebellum on fear-related, classical heart-rate conditioning in goldfish.

**Methods:**

The effects of microinjection of the anesthetic agent lidocaine into the cerebellum on fear conditioning were investigated in goldfish. The fear conditioning paradigm was delayed classical conditioning with light as a conditioned stimulus and electric shock as an unconditioned stimulus; cardiac deceleration (bradycardia) was the conditioned response.

**Results:**

Injecting lidocaine into the cerebellum had no effect on the base heart rate, an arousal/orienting response to the novel stimulus (i.e., the first presentation of light), or an unconditioned response to electric shock. However, lidocaine injection greatly impaired acquisition of conditioned bradycardia. Lidocaine injection 60 min before the start of the conditioning procedure showed no effect on acquisition of conditioned bradycardia, indicating that the effect of lidocaine was reversible.

**Conclusions:**

The present results further confirm the idea that the cerebellum in teleost fish, as in mammals, is critically involved in classical fear conditioning.

## Background

In addition to its well-known roles in motor coordination and discrete motor learning [1], the cerebellum is involved in emotion and its learning in mammals [2,3]. Brain mechanisms of fear learning are one of the well-documented neural substrates of emotional learning [3-5]. Besides the amygdala, of which emotion roles have been intensively studied, the cerebellum has also been demonstrated to play a critical role in simple classical fear conditioning in mammals [3,6,7]. The vermal part of the cerebellum in mammals has been suggested to be homologous with the corpus cerebelli (CC) in fishes [8] and have been implicated in fear-related behaviors [2,6]. The integrity of the amygdala and cerebellum is required for normal performance of classical fear conditioning [9]. Fear conditioning-related synaptic changes in the cerebellar circuit have also been reported in rats [10]. In teleostean cerebellar Purkinje cells, both LTP and LTD, that have similar cellular mechanisms to those in mammarian Purkinje cells, have been observed in mormyrid fish [11]. This suggests that the cerebellar synaptic plasticity in classical conditioning is shared by mammals and fish.

The medial telencephalic pallium (MP) in teleost fish has been suggested to be homologous with the amygdala in mammals [12,13], and lesions in this region impair active avoidance learning in goldfish [14], which is believed to be based on the mediational state of fear [13,15]. On the other hand, classical aversive conditioning, in which an autonomic bradycardic response is conditioned, is spared even after ablation of the entire telencephalon in goldfish [16].

Cerebellar inactivation greatly impairs simple classical fear conditioning in goldfish [17,18]. It is interesting to note that mammals and fishes may share common brain mechanisms for fear-related emotional learning. Revealing the differential roles and interactions of the telencephalon and cerebellum in fear-related conditioning in fish should shed light on the evolution of fear and underlying neural mechanisms.

Lines of evidence have been accumulating regarding the function of the telencephalon, which has been suggested to be partly homologous to the limbic system in the mammalian brain, for emotional learning in fish [13,15]. However, the role of the cerebellum in emotional learning in teleost fish is poorly understood, whereas the cerebellum obviously shares major intrinsic circuitry, as well as afferent and efferent connections with other brain parts, with those in the mammalian cerebellum [19-22].

Until date, lesion studies have been mainly conducted to investigate the involvement of the cerebellum in fear-related classical conditioning in fish [17,18]. Only one study concerning the effect of reversible inactivation of the cerebellum on classical aversive learning is available [17]. In this study, cerebellar activity was partly and temporarily inactivated during the conditioning procedure by localized cooling of the CC. Evaluation of the effects of chemical agent administration on neural activity in the cerebellum is required for further investigation of the role of cerebellar circuitry in emotional learning in fish.

Sacchetti et al. [9] have noted that one difficulty while searching for the site of memory storage for fear learning is that the blockade of a focused site also affects some other behaviors. However, in the case of goldfish cerebellum, partial ablation apparently does not affect general activity as much as specific motor performance [23]. Although the cerebellum is suggested to be involved in other cognitive functions, such as spatial learning [18], goldfish cerebellum can be a suitable model for studying the neural mechanisms of classical emotional conditioning. CC of the goldfish is easily accessible, enabling injection of agents into this brain region during an acute conditioning session.

In the present study, we examined the effect of local administration of the anesthetic agent lidocaine into the cerebellum on fear-related, classical heart-rate conditioning in goldfish. Local administration of lidocaine into the brain has been used for reversible lesioning of the injected site [24,25].

## Methods

### Animals

Commercially obtained goldfish (*Carassius auratus*), 72-135 mm in body length, were kept in our laboratory at a water temperature of 23-26°C for more than 1 month before use. The light/dark cycle of the room was 14 h/10 h, and all experiments were performed during the light period. All animal experiments were conducted under the Guidelines for Animal Experimentation, Hiroshima University.

### Application of lidocaine and conditioning experiments

Goldfish were anesthetized in 0.015% tricaine methane sulfonate (MS-222, Crescent Research Chemicals, Phoenix, AZ, USA), and the neuromuscular blocking agent d-tubocurarine chloride (5 μg/g body weight; Nacalai tesque, Tokyo, Japan) dissolved in saline was injected intraperitonially. A window (about 5 × 5 mm) was opened in the cranium over CC, and the fat tissue covering the dorsal part of CC was carefully removed with forceps. The fish were gently restrained between a pair of urethane foam pieces in the conditioning chamber, and the gills were irrigated with aerated water supplied through a tube inserted in the mouth. The water surface in the chamber was kept just below the window opened in the cranium. In the first experiment, goldfish were assigned to three groups; control (n = 10), vehicle (n = 10), and lidocaine (n = 10). In the vehicle and lidocaine groups, a 33-G tapered needle (TN-3305, Terumo, Tokyo, Japan) connected to a microinjector (IM-9B, Narishige, Tokyo, Japan) through a polyethylene tube was inserted into CC at a depth of 1 mm from the dorsal surface, using a manipulator (MM-3, Narishige). The fish were then allowed to adapt for 1.5 h before the conditioning procedure began. In the control group, the procedure was the same as that in the vehicle and lidocaine groups, except for insertion of the needle.

The conditioning procedure consisted of three sessions: habituation (10 trials), acquisition (20 trials), and extinction (15 trials). The conditioning stimulus (CS) was a 5.1-s illumination of a green light-emitting diode (LED) placed on the right side of the head. The unconditioned stimulus (US) was a 20-V electric shock applied to the trunk through a pair of silver plates (10 × 10 mm). The intertrial interval was 60 s. CS was presented alone during the habituation and extinction sessions. In the acquisition session, the last 0.1 s of CS overlapped with the presentation of US. Heart beats were recorded by a photocardiography technique [26] in which the cardiac activity was optically and noninvasively monitored. The magnitude of the conditioned bradycardic response was quantified as the bradycardia index. The bradycardia index was calculated according to Yoshida et al. [26]. Briefly, the heart beat frequency during a 5-s period after onset of CS was subtracted from the heart beat frequency during a 5-s period before onset of CS (pre-CS heart beat frequency). The value obtained was then divided by the pre-CS heart beat frequency, yielding the bradycardia index. If no heart beat occurred during CS, the bradycardia index was 1. If tachycardia occurred in response to CS, the index was a negative value.

To observe the immediate effect of lidocaine application on acquisition performance, injections were started just after the end of the tenth trial of the habituation session and ended before the fourth trial of the acquisition session. In the lidocaine group, artificial cerebrospinal fluid (ACF; NaCl, 140 mM; KCl, 3 mM; CaCl_2_, 3 mM; MgSO_4_, 2 mM; HEPES, 10 mM; pH = 7.5) containing 1% brilliant blue 6B (Nacalai tesque) and 1% lidocaine hydrochloride (Alexis Biochemicals, Plymouth Meeting, PA, USA) was injected. In the vehicle group, ACF containing 1% brilliant blue was injected. The total injection volume was 680 nl. It took about 5 min to complete the injection. The needle was withdrawn immediately after the injection. After completing the conditioning experiment, the fish were deeply anesthetized with MS-222 and decapitated. Because no significant differences were observed between the control and vehicle groups, the vehicle group was not used in the following experiment.

In the second experiment in which recovery from the effect of lidocaine was examined, goldfish were assigned to three groups: control (n = 10), 0 min (n = 10), and 60 min (n = 10). The surgical procedure, injection apparatus, CS and US presentations, and quantification of the conditioned response were the same as those described above. Control fish underwent a conditioning procedure including habituation (10 trials) and acquisition (20 trials) sessions after a 2-h adaptation period in the conditioning chamber. Goldfish in the 0-min group received an ACF injection containing 1% brilliant blue and 1% lidocaine hydrochloride after a 2-h adaptation period and just before the habituation session began. Goldfish in the 60-min group received an ACF injection containing 1% brilliant blue and 1% lidocaine hydrochloride after a 1-h adaptation period and were then allowed to recover for another 1 h before commencing the conditioning procedure. The total injection volume in the 0- and 60-min groups was 340 nl, and it took 2.5 min to complete the injection. The total injection volume was a half of that in the first experiment. That was because the recovery should be as quick as possible, since prolonged experiment period may well cause deterioration of physiological conditions of the fish. After completing the experiment, the fish were deeply anesthetized with MS-222 and decapitated.

### Statistics

The Freedman test was used to compare base heart rates among the three stages (tenth habituation trial as well as fifth and twentieth acquisition trials) in each group. The Steel test was used to compare the base heart rates and bradycardia indices (i.e., the magnitude of conditioned responses) in the vehicle and lidocaine groups with those in the control group in the first experiment. Steel test was also used to compare bradycardia indices in the 0-min and 60-min groups with that in control group in the second experiment. The Wilcoxon matched-pairs signed-ranks test was used to analyze the performance of acquisition and extinction in relation to the habituation level in each group. Differences were considered to be significant when p < 0.05.

## Results

### Microinjection of lidocaine

**Figure 1:**
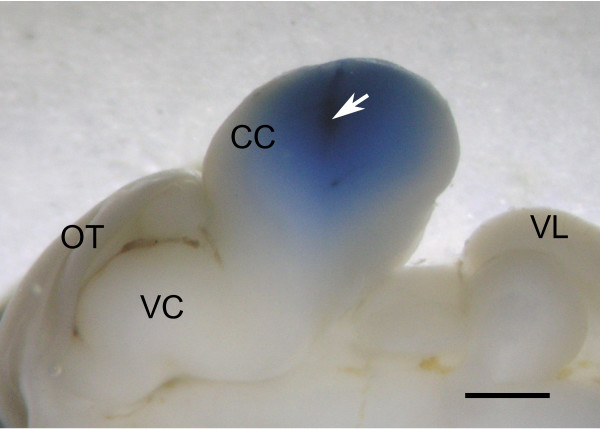
**Photograph of the sagittal plane of goldfish brain cut at midline showing the diffusion of brilliant blue in the injected solution**. Arrow indicates injection site. CC, corpus cerebelli; OT, optic tectum; VC, valvula cerebelli; VL, vagal lobe. Scale bar = 1 mm.

Figure 1 shows the photomicrograph of a sagittally cut plane of goldfish brain subjected to lidocaine injection into CC. The diffusion of brilliant blue appeared to be restricted to within CC, although the extent of diffusion varied among individuals. Because the molecular weight of brilliant blue (992.8) is larger than that of lidocaine (234.3), lidocaine may have diffused outside the area of brilliant blue diffusion. However, because lidocaine was partially metabolized, it was difficult to precisely estimate the effective area of the injected anesthetic agent.

**Figure 2:**
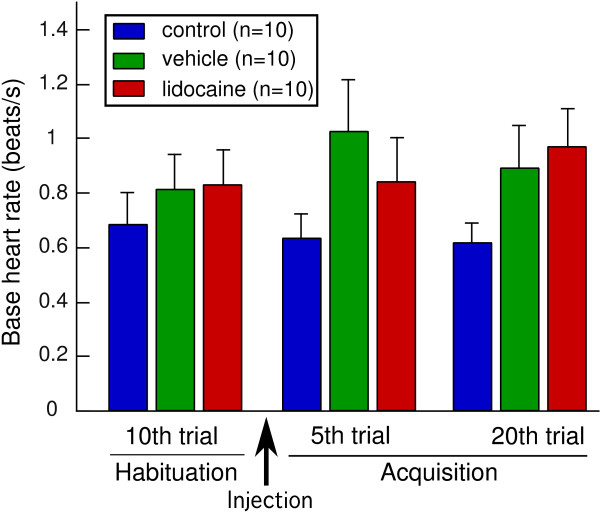
**Effects of lidocaine injection on base heart rate during habituation and acquisition sessions**. During the acquisition session, base heart rates in the fifth trial (just after completion of the injection) and twentieth trial (about 15 min after the injection) are shown. There were no significant differences in base heart rates between these trials in each group. Base heart rates in the vehicle and lidocaine-injected groups were not significantly different from those in the control group in each trial.

To examine whether lidocaine application affected the base heart rate of goldfish, the average heart rate during a 5-s pre-CS period of the tenth habituation trial, which was the last trial before lidocaine injection, was compared to that in the fifth acquisition trial, which was the first trial after completing the lidocaine injection, and that in the twentieth acquisition trial. There were no significant differences in the groups among these three trials (Friedman test, p > 0.05) (Figure 2). The Steel test revealed that the base heart rates in both the vehicle and lidocaine-injected groups were not significantly different from that in the control group even after injection (p > 0.05) (Figure 2).

### Effects of lidocaine application on conditioning

**Figure 3:**
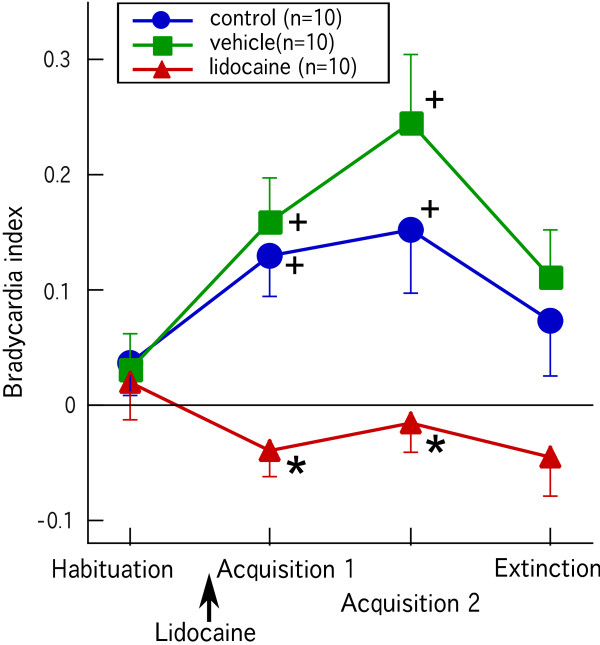
**Effects of lidocaine injection into CC on acquisition of a conditioned bradycardic response**. Average bradycardic responses in the habituation session, the first 10 trials of acquisition session (Acquisition 1), the last 10 trials of the acquisition session (Acquisition 2), and the extinction session are shown. * denotes significant differences from the control group. + denotes significant differences from the habituation level in each group.

Although the goldfish responded to a novel visual stimulus, i.e., the first CS presentation, with cardiac deceleration, this arousal and/or orienting response disappeared within a few trials. This observation was consistent with the findings of a previous report [15]. Figure 3 shows the effect of applying lidocaine to CC on acquisition of a conditioned bradycardic response. In the control and vehicle groups, the average bradycardia indices in the first and last 10 trials of the acquisition session were significantly larger than those during the habituation session (Wilcoxon matched-pairs signed-ranks test, p < 0.05) (Figure 3). In contrast, lidocaine-injected goldfish did not develop a conditioned bradycardic response (Figure 3). There were significant differences in bradycardia indices in the first and last 10 trials in the acquisition session between the lidocaine-injected and control groups (Steel test, p < 0.05), whereas the level of acquisition in the vehicle group was not significantly different from that in the control group (Steel test, p > 0.05) (Figure 3). This observation shows that the effect of lidocaine was apparent throughout the acquisition session from immediately after injection to the end of the session. Repeated presentations of CS alone (extinction session) partly diminished the conditioned response in the control and vehicle groups (Figure 3). The average bradycardia indices in the last 10 trials of the extinction session showed no significant differences compared to the habituation level in all groups (Wilcoxon matched-pairs signed-ranks test, p > 0.05) (Figure 3). Because there were no differences between the control and vehicle groups, the vehicle group was not used in the following experiment.

**Figure 4:**
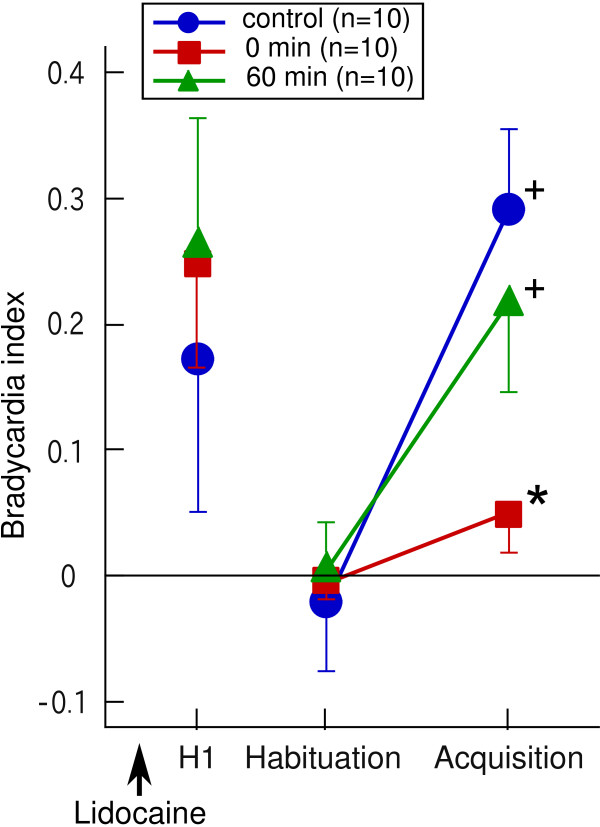
**Reversible effect of lidocaine injection into CC on acquisition of a conditioned bradycardic response**. Lidocaine was injected before starting the habituation session. Arousal/orienting responses to the first presentation of the conditioned stimulus (H1) are also shown. * denotes significant difference from the control group. + denote significant differences from the habituation level in each group.

Because lidocaine is an anesthetic agent with a short duration (less than 1 h) [25], cerebellar functions should be restored after diffusion and metabolism of the agent. Thus, in the second experiment, recovery from the lidocaine injection was examined. Figure 4 shows the reversible effect of lidocaine application to CC on heart rate conditioning and the effect on the arousal response to the first CS presentation. In the group in which the conditioning procedure started just after the lidocaine injection (0-min group), there was no significant conditioned bradycardic response (Figure 4). Furthermore, the average bradycardia index in the last 10 trials of the acquisition session in the 0-min group was significantly smaller than that in the control group (Steel test, p < 0.05) (Figure 4). In contrast, the goldfish in which the conditioning procedure was started 60 min after the lidocaine injection (60-min group) developed a significant conditioned response during the acquisition session (Wilcoxon matched-pairs singed-ranks test, p < 0.05) (Figure 4). The level of the conditioned response in the 60-min group was not different from that in the control fish (Figure 4).

**Figure 5:**
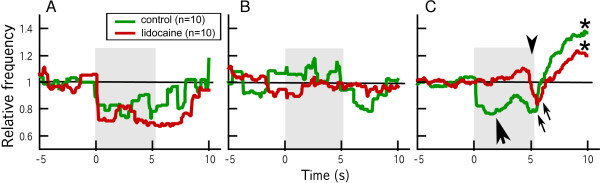
**Responses of heart rate to CS and US**. A. Response to the first presentation of CS in control and lidocaine-injected goldfish. Both control and lidocaine-injected fish showed an arousal/orienting response. B. The tenth trial of the habituation session. Note that there was no cardiac deceleration in response to CS. C. Averages of the cardiac responses in the first 10 trials during the acquisition session. Arrowhead denotes the time of US presentation. Large arrow denotes conditioned bradycardic response in control fish. Both control and lidocaine-injected fish showed unconditioned bradycardic responses (small arrows) followed by tachycardic responses (asterisks) to US. Average instantaneous frequencies in relation to the average levels during the 5-s period before CS presentations are shown.

In this experiment, lidocaine was injected before the habituation session. Lidocaine injection did not alter the arousal response to the first presentation of CS in both 0-min and 60-min groups (Figure 4, 5A), indicating that the pathway from the visual sensation of CS to the control center for heart beat was intact in lidocaine-injected fish. The arousal response declined quickly as the habituation session proceeded, and the response almost disappeared in the last trial of the habituation session (Figure 5B).

To examine the possibility that development of the conditioned response in lidocaine-injected fish was impaired because of decreased sensitivity to US (electric shock), cardiac responses to US were compared between the control and 0-min groups. A normal unconditioned response consisted of two components: cardiac deceleration (bradycardia) immediately after US presentation, followed by cardiac acceleration (tachycardia) that lasted a relatively long period (Figure 5C). Thus, only the first bradycardic component was conditioned in the present procedure. We found no substantial differences in the bradycardic and tachycardic components of the unconditioned response between the control and lidocaine-injected groups (Figure 5C).

## Discussion

We found that the reversible lesion of CC by injection of lidocaine into CC markedly impaired acquisition of conditioned bradycardia during classical fear conditioning in goldfish. The present result strongly supports the idea that CC plays a crucial role in fear conditioning in fish [17,18].

Microinjecting solutions containing various substances into the goldfish cerebellum is a useful method to investigate the neuronal substrates underlying classical fear conditioning. The anatomical accessibility of CC, which is situated just beneath the dorsal cranium, and the relatively quick acquisition of a conditioned cardiac response during the classical fear conditioning paradigm are advantages of the present system. Although a precise determination of lidocaine diffusion was difficult, it appeared that diffusion was limited within CC. CC of goldfish protrudes dorsally, which may be the reason for restriction of diffusion within this part of the brain. Because the ACF injection containing brilliant blue did not affect classical fear conditioning, injection of a small volume (680 nl) of the solution itself had little effect on cerebellar function, and we found that lidocaine injection into the cerebellum did not alter the base heart rate. This result also supports the idea that the effective concentration of lidocaine did not reach other brain areas such as the medulla, which is the regulatory center for heart rate.

Lidocaine has been used for local and temporary anesthesia of the central nervous system [25,27] as well as the peripheral nervous system [28,29] in fish. Because the effective duration of lidocaine is short [25], it was first injected just after the habituation session to test the effect of immediate inactivation of cerebellar activity on acquisition of a conditioned bradycardic response. We found no significant development of a conditioned response in lidocaine-injected goldfish that underwent 20 trials of paired presentations of CS and US. Approximately 15 paired presentations are enough for the majority of goldfish to acquire a maximum conditioned response using a conditioning procedure identical to that in the present experiment [17]. In the situation of delay classical eyeblink (eye-retraction) conditioning in goldfish, in which the cerebellum is also involved, it has been reported that the conditioned response to the CS presentation is accurately timed to the onset of the US [18]. On the other hand, in the present heart-rate conditioning, peak of the amplitude of the conditioned response appeared just after the onset of the CS (see Figure 5).

In the present experiment, we found that the average bradycardia index, i.e., the magnitude of the conditioned response, in the first 10 trials of the acquisition session was significantly greater than the habituation level in the control and vehicle groups. However, there was no development of conditioned bradycardia in the first and latter half of the acquisition session in the lidocaine-injected group. Therefore, lidocaine seemed to be effective immediately after the injection and maintained its effectiveness throughout the acquisition session, which lasted for about 25 min. There was no significant effect on the acquisition of the conditioned response when lidocaine was injected 1 h before commencement of the conditioning procedure. Thus, the small amount of lidocaine applied to CC appeared to diffuse and get metabolized within 1 h.

Because cardiac deceleration, which is an arousal and/or orienting response to a novel visual stimulus (LED light) [30,31], was apparent in lidocaine-injected fish, it is unlikely that the applied lidocaine affected the sensory pathway mediating the visual stimulus. Furthermore, lidocaine-injected fish showed an unconditioned response to US (electric shock), similar to that in control fish. These results show that the failure in acquisition of the conditioned bradycardia in lidocaine-injected fish was not due to sensory disruption or impairment in the center directly regulating heart rate.

The present results suggest that the cerebellum plays roles in sensory association of CS and US and/or memory storage. The immediate and reversible effect of lidocaine would be useful in further experiments in which cerebellar activities could be temporarily stopped at an appropriate time to investigate the role of the cerebellum in classical fear conditioning. In mammals, the cerebellum is critically involved in emotional learning, especially fear-related conditioning [6,7]. Lesion studies in rats and rabbits have revealed that the cerebellar vermis plays an important role in classical fear conditioning [6,7]. In rats, fear-conditioning-related changes in synaptic transmission onto cerebellar Purkinje cells have also been reported [10]. Thus, the cerebellum, together with the amygdala, is one of the network regions involved in fear and its learning [9].

Histological and physiological studies have suggested that the cerebellar inputs and outputs in teleost fish are similar to those in mammals [21,22]. Furthermore, intrinsic cerebellar circuitry in teleost fish shares basic features with that in mammals [19,20]. One major exception is the output neurons of the cerebellar cortex [32,33]. In teleosts, efferent output from the cerebellar cortex is conducted by eurydendroid cells but not Purkinje cells as in mammals. However, the eurydendroid cells are suggested to be displaced cerebellar nuclei neurons, which are cerebellar efferent neurons in tetrapods [19,33,34]. In goldfish, the cerebellum has shown to have various efferent targets including the diencephalon and the medulla [35]. Given that the teleost cerebellum has anatomical and functional homology with the mammalian cerebellum [12,32], teleost fish can be a useful model for studying neuronal substrates of emotional learning involving the cerebellum.

The amygdala is critically involved in classical fear conditioning in mammals [36-38]. It has been recently demonstrated that the MP region of the telencephalon in teleost fish is homologous with the amygdala in tetrapods [13], and lesions in the MP region selectively impair active avoidance learning in goldfish [14,39]. Impaired active avoidance learning in MP-lesioned fish is believed to be because of a disability in acquisition of a mediational state of fear [13,15], whereas classical fear conditioning of autonomic response (cardiac deceleration) is spared in goldfish subjected to ablation of the entire telencephalon [16]. It may be that the telencephalon, namely the MP region, is essential for fear-related learning that involves instrumental components, whereas the cerebellum is critical for the classical part of fear learning. Neuronal networks subserving emotion and its learning may be more conserved over vertebrate evolution than previously thought [13,18,40]. It has been reported that sensory inputs are conveyed to the different areas of the CC depending on modalities [41]. Neural responses to visual stimuli have observed mainly in the medial part of the CC [41]. More localized application of lidocaine to the CC may reveal the relationships between substructure and fear conditioning involving visual cues. In addition, surgical ablation of CC does not severely affect the general activity of goldfish [18,23], and cerebellar involvement in spatial learning has been reported in goldfish [18]. Although it is obvious that the integrity of the cerebellum is required for fine control of motor performance [23,25,42], higher cognitive processing may be another major role of the cerebellum in teleost fish.

## Conclusions

The present results support the idea that the cerebellum in teleost fish, as in mammals, is critically involved in classical fear conditioning. Although it is obvious that the cerebellum is required for control of motor performance, higher cognitive processing may be another major role of the cerebellum in teleost fish.

## Competing interests

The authors declare that they have no competing interests.

## Authors' contributions

MY designed the study, performed the statistical analysis and drafted the manuscript. RH carried out the physiological, histological and behavioral studies. All authors read and approved the final manuscript.
